# Analysis and Forecasting Incidence, Intensive Care Unit Admissions, and Projected Mortality Attributable to COVID-19 in Portugal, the UK, Germany, Italy, and France: Predictions for 4 Weeks Ahead

**DOI:** 10.3390/bioengineering8060084

**Published:** 2021-06-11

**Authors:** Kathleen Carvalho, João Paulo Vicente, Mihajlo Jakovljevic, João Paulo Ramos Teixeira

**Affiliations:** 1 Research Centre in Digitalization and Intelligent Robotics (CeDRI)—Instituto Politecnico de Braganca, 5300-253 Bragança, Portugal; carvalho.kath@gmail.com (K.C.); joaopaulovicen@gmail.com (J.P.V.); 2 Campus Cornélio Procópio, Federal Technological University of Paraná, Paraná 86300-000, Brazil; 3 Department of Global Health Economics and Policy, Faculty of Medical Sciences, University of Kragujevac, 34000 Kragujevac, Serbia; sidartagothama@gmail.com; 4 Institute of Comparative Economic Studies, Hosei University, Tokyo 102-8160, Japan; 5 Applied Management Research Unit (UNIAG), Instituto Politecnico de Braganca, 5300-253 Bragança, Portugal

**Keywords:** time series prediction, ANN forecasting, new coronavirus, COVID-19 prediction cases, COVID-19 prediction deaths, COVID-19 prediction ICU, COVID-19 vaccination, COVID-19 in Europe, COVID-19 in Israel, COVID-19 wearing of face mask

## Abstract

The use of artificial neural networks (ANNs) is a great contribution to medical studies since the application of forecasting concepts allows for the analysis of future diseases propagation. In this context, this paper presents a study of the new coronavirus SARS-COV-2 with a focus on verifying the virus propagation associated with mitigation procedures and massive vaccination campaigns. There were two proposed methodologies in making predictions 28 days ahead for the number of new cases, deaths, and ICU patients of five European countries: Portugal, France, Italy, the United Kingdom, and Germany. A case study of the results of massive immunization in Israel was also considered. The data input of cases, deaths, and daily ICU patients was normalized to reduce discrepant numbers due to the countries’ size and the cumulative vaccination values by the percentage of population immunized (with at least one dose of the vaccine). As a comparative criterion, the calculation of the mean absolute error (MAE) of all predictions presents the best methodology, targeting other possibilities of use for the method proposed. The best architecture achieved a general MAE for the 1-to-28-day ahead forecast, which is lower than 30 cases, 0.6 deaths, and 2.5 ICU patients per million people.

## 1. Introduction

The pandemic declaration by the World Health Organization (WHO) in March 2020 presented to the world the great challenge of controlling the disease caused by the new coronavirus. The race between nations for the study and approval of medicines and vaccines has started since that time. This resulted in achieving public and private investments to streamline the stages of development [1].

In the research context, the effectiveness of drugs that are already existent in the market as possible treatments was evaluated [2]. However, despite the insistence of some political fronts, these drugs have been under discussion for too long, even though their ineffectiveness has been proven to cause long-term damage to people who used them without medical advice. A controversial example is hydroxychloroquine, used in the treatment of lupus, malaria, and rheumatoid arthritis. Further tests have shown the drug to be ineffective in reducing the risk of patients having respiratory problems and subsequently being placed on ventilators [3]. To this date, the treatment of COVID-19 has been carried out by the individual assessment of each patient, without a single treatment protocol that has yet been established.

The advent of several vaccines happened in the second semester of 2020. According to WHO correspondent Dr. Cristina Toscano in an interview, despite the suspicion about the effectiveness of immunizing (vaccine) due to the short time spent on studies and tests, the rapid development was carried out with both the help of previous research on coronaviruses and innovative technologies—whose development has lasted two decades—and the increased focused funding received for the last couple of years. 

Some authors have already proposed the use of time series forecasting applied to prediction data about coronavirus propagation. In Borghi et al. [4], the multilayer perceptron network was trained with thirty countries’ data using the previous 20 days of new cases and deaths, along with the accumulative number of cases and deaths to predict the accumulative cases and deaths for six days ahead. Similarly, the methodology proposed by Oliveira et al. [5] uses a combination of eight countries’ data of the accumulative number of cases and deaths applied to two training algorithms, i.e., Levenberg–Marquardt and the resilient propagation, to predict seven days of accumulative cases and deaths for Portugal, Brazil and the United States. With a focus more on using data of Asian countries for training, Tamang et al. [6] used three different analyses to propose a method to predict the accumulative number of cases for ten days in India, the United States, France, and the UK, while Wieczored et al. [7] used a deep architecture with the NAdam training model to forecast fourteen days for several countries and regions of accumulative number of cases. Meanwhile, the methodology proposed in this paper predicts data for twenty-eight days for the daily number of new cases, deaths, and ICU patients—not accumulative values as in the previous methods—and it uses information about the wearing of masks and the vaccination situation in each country.

Within the context of mass immunization in several countries, this article presents a study of the spread of COVID-19 in Europe and includes an analysis of the prediction of new cases, deaths, and ICU patients, based on two analytical approaches applied to artificial neural networks. The algorithms used adopts a direct forecasting approach for several days (n is the next day): n to n + 6, n + 7 to n + 13, n + 14 to n + 20, and n + 21 to n + 27 days ahead, and a recursive approach for a range of 28 days, in order to compare the results of the approaches. Both sets serve to analyze the spread of the virus associated with methods of mitigation and mass immunization.

The description of methodologies used to predict new cases, deaths, and ICU patients are detailed in Section 2. The normalization of the data is carried out per million people of each country to reduce the discrepancies of absolute numbers, thus allowing for a clearer analysis as to the effectiveness of the mitigation methods. Moreover, a moving average of seven days is applied to the data in order to minimize errors caused by underreporting on weekends.

In addition, a case study is presented on new infections and deaths in Israel and the consequences of quick vaccination. It is important to highlight that vaccination in Israel has been carried out exclusively with the vaccine developed by a partnership between BioNTech and the Pfizer group; this case facilitated the percentage analysis of the efficacy of the immunological window [8].

The use of ANN for time series forecasting is recommended since it can work with independents inputs as quantitative and qualitative data at the same network with high accuracy. The chosen auxiliary input data used in this work are associated with virus propagation variables, such as the mandatory wearing of masks and the progress of vaccination campaigns.

For political and social decisions in a pandemic situation, the accuracy of forecasting can help in evaluating different scenarios according to mitigation procedures. With this in mind, the main contribution of this work is to provide a prediction methodology based on daily data (not accumulative) for almost a month, accounting for important variables such as the number of new cases, deaths and ICU patients, while considering the numbers of the previous 21 days of cases, deaths, and ICU patients, the percentage of vaccination, and the wearing of masks.

Following this introduction, this manuscript is organized as follows. Section 2 presents the material and methodologies with an analysis of the virus propagation in six countries—which includes a case study of Israel. Section 3 follows with the results of the mass vaccination campaign, the results of forecasting new cases, deaths, and ICU patients for the five countries using different approaches, and the case study of Isreal. The same section includes the discussion and comparison of the different methodologies and the results. The conclusions are listed in Section 4.

## 2. Materials and Methods

The present study aimed to analyze and forecast the waves of new cases, deaths, and ICU patients of COVID-19. The time series data were normalized per million people of each country in order to analyze the virus propagation independently of the size of the country. Moreover, two methodologies of forecasting were proposed, defined by (1) the recursive approach and (2) the N approach. Both forecasting methodologies use previous data of 21 days before (n − 21: n − 1) in relation to the number of new cases, number of deaths, number of ICU patients, information about the mandatory wearing of masks on the previous day (n − 1), and the percentage of immunized people at least with the first dose of a vaccine.

The models were used to predict the number of new cases, deaths, and ICU for next days. In the recursive approach, the previous 21 days are used to predict only one day ahead (n), then the data are updated with predicted numbers to preview the next day, in a recursive iteration, until the desired number of days ahead. With the N approach, the ANN model uses the same previous 21 days, but the output is the predicted numbers for a range of days ahead, i.e., from n (next day) to n + 7, n + 14, and n + 21, using four outputs (one for each week ahead), and recursively determine the 7 days of the week.

### 2.1. Data Collection Source

For the development of this work, the established database to train, validate, and test the ANN includes qualitative and quantitative data that were associated with the term between 25 January 2020 and 14 March 2021. The quantitative data used are related to the daily new cases and deaths, the daily number of ICU patients, and the percentage number of people immunized with the first dose of a vaccine. On the other hand, the qualitative data used in this study are associated with the mitigation procedures, specifically the mandatory wearing of masks. The data about lockdown in the countries were not available to be applied as an input, since the restrictions were defined with different levels and conditions for each country and sometimes with differences.

The proposed forecasting methodology of COVID-19 new cases, deaths, and ICU patients was based on analyses of five European countries, namely Portugal, France, Italy, UK, and Germany. However, at the beginning of the tests, Israel was also included, since at the time, it was the country with a higher level of immunization through a mass vaccination campaign.

The data gathered were oriented mainly by the Worldometer and Our World in Data websites [9], both used as references by media such as Johns Hopkins CSSE, Financial Times, The New York Times, and Business Insider. The websites combine the information provided from the official media platforms of each country to simplify analyses on the same platform [10,11,12,13,14,15].

For the information about the wearing of masks, the chosen source was the website Masks4All [16], which reports when the wearing of masks started being mandatory in public places in each country. The information input to the ANN assumes a value 1 from the date when the masks began being worn as a mandatory precaution, and 0 before that date.

### 2.2. Data Processing

The absolute numbers of new cases, deaths, and ICU patients are directly related to the size of the country’s population. Therefore, it is necessary to normalize the daily numbers by a standard for all countries. The normalization method was determined per million people, calculated by dividing each daily number by the population of the respective country (in millions without rounding). In order to reduce oscillatory data caused by weekend underreporting, a moving average of seven days was also applied

The accumulative vaccination data used as input refer to the last day before the prediction and were defined as a percentage of the population of each country that received at least one shot of vaccination. Finally, the qualitative data on the wearing of masks do not require normalization since the data are binary and refer to the last day before the first prediction (n − 1). The inputs of each ANN architecture are composed of 70 nodes, of which 5 are for country binary identification and the last 65 for all quantitative and qualitative data, corresponding to the 21 previous days (n − 21 to n − 1) for cases, deaths, and ICU numbers, plus the wearing of masks and the vaccination number on the previous day (n − 1).

The dataset were divided for training and validation and for testing procedures. The data of all countries under study, related to 25 January 2020 until 14 February 2021, were combined and divided randomly in 75% for training and 25% for validation. During the training stage, the ANN learned the time series behavior, adjusting the ANN weights and using only the training data in a sequence of iterations. The validation dataset was used at each iteration to control the ANN performance with a different dataset, and the ANN stops training when performance stops increasing. This procedure avoids having the ANN overfit the training dataset, which would risk losing generalization capacity. The data from 15 February to 14 March 2021 were separated for the test dataset. The test dataset was not seen by the model during the training procedure and was used only to evaluate the model performance.

### 2.3. Recursive Approach

**Figure 1:**
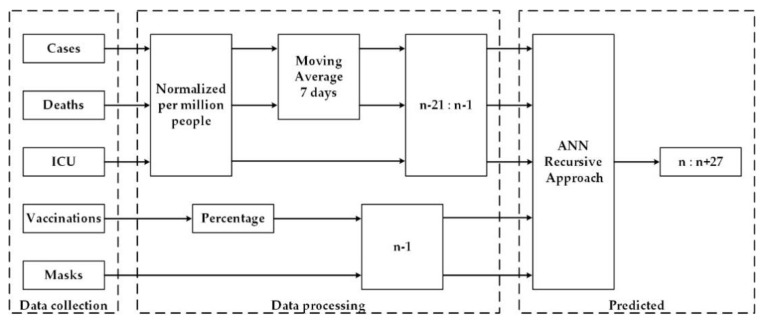
Prediction diagram for the 3 ANN (cases, deaths and ICU) using the recursive approach.

In this approach, illustrated in Figure 1, the daily numbers of cases, deaths, and ICU patients were forecasted for the period between 15 February and 14 March of 2021 (test dataset) for Portugal, France, Italy, the UK, and Germany. The multilayer perceptron ANN architecture was used as the computational tool for the prediction of the mentioned data. Since the behavior of the time series of deaths, ICU and cases tend to have a delay between each other, as one ANN was used for each of this time series but the same for all countries. In any case, the input and methodology were the same for the three ANNs.

The forecasting methodology of this approach consisted of predicting the new number of cases, deaths, and daily ICU patients by one day ahead, related to the data used as input. The predictions were used in the next iterations for the next days.

Several architectures’ combination were experimented using the training/validation dataset. For the three ANNs used to forecast, the best topology has only one hidden layer with different numbers of neurons in a hidden layer and has activation functions in the hidden and output layers. All experiments used the Levenberg–Marquardt training algorithm [17]. The best architecture for each situation is shown in Table 1, as the one adopted for this approach.

### 2.4. N Approach

**Figure 2:**
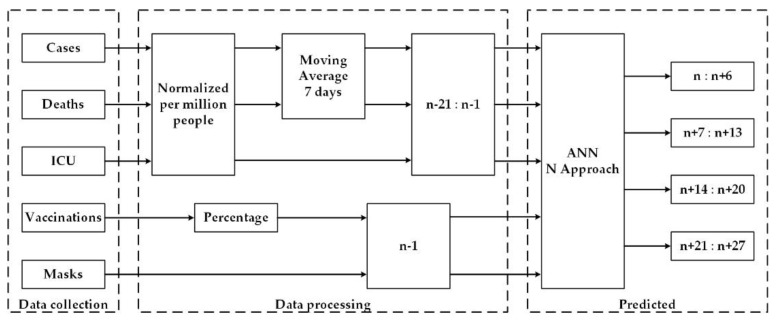
Prediction diagram using the N approach.

The N approach proposes predicting the new cases, deaths, and daily ICU patients of the same period of the previous approach, but in this case, the forecasting total vector is divided by week. Its scheme is shown in Figure 2.

The forecasting methodology of this approach consisted of forecasting new cases, deaths, and ICU patients (one ANN for each), for days n (first day), n + 7, n + 14, and n + 21. The input for each ANN had 70 nodes (detailed in Section 2.2) to code the country, new cases, deaths, ICU patients, vaccination, and mandatory wearing of masks. Each ANN had 4 outputs. The prediction was made in a sequence of seven, which resulted in the forecast of the following days for each output of the ANN: n to n + 6, in node 1; n + 7 to n + 13, in node 2; n + 14 to n + 20, in node 3; and n + 21 to n + 27 in node 4. For the present experiment, the following days were predicted in each of the four nodes:

- The 1st day (n): 15 February 2021 to 21 February 2021
- The 8th day (n + 7): 22 February 2021 to 28 February 2021
- The 15th day (n + 14): 1 March 21 to 7 March 2021
- The 22nd day (n + 21): 8 March 2021 to 14 March 2021

Similar to the recursive approach, three separate ANNs were used in forecasting using only one hidden layer; different numbers of neurons were used in the hidden layer, with different activation functions in the hidden and output layers, and the Levenberg–Marquardt was also used as the training algorithm [17]. The best architecture for each situation is presented in Table 2.

### 2.5. Error Measurement

To compare the recursive and N approaches, the mean absolute error (MAE) for each methodology was calculated by Equation (1) to predict new cases, deaths, and ICU patients for the following week cases:

- Between 15 February 2021 and 21 February 2021 (next week)
- Between 22 February 2021 and 28 February 2021 (two weeks ahead)
- Between 1 March 2021 and 7 March 2021 (three weeks ahead)
- Between 8 March 2021 and 14 March 2021 (four weeks ahead)
- Between 15 February 2021 and 14 March 2021 (next month)

(1) $$\mathrm{MAE}=\frac{1}{n}\overset{n}{\underset{i=1}{\sum }}\left|Real_{i}-Predicted_{i}\right|$$

MAE was used because once the time series is normalized per million people, a simple multiplication of MAE by the number of million people of each country gives the number of cases/deaths/ICUs. This daily information is objective and intelligible in showing the distance of the real and predicted numbers.

The last error analysis is based on the mean absolute percentage error (MAPE) in order to evaluate the accuracy of the proposed forecasting methodology by the relative error, between the real and predicted values, as presented in Equation (2).

(2) $$\mathrm{MAPE}=\frac{100}{n}\overset{n}{\underset{i=1}{\sum }}\left|\frac{Real_{i}-Predicted_{i}}{Real_{i}}\right|$$

## 3. Analysis, Results, and Discussion

The analysis of the cases and deaths for the European countries and Israel is presented in the following subsection. Since the behavior of Israel’s time series is different from the ones of the European countries, they were excluded from the dataset to train the prediction model, and consequently there were no forecasting results. Nevertheless, some analysis of the time series of Israel is presented in Section 3.2 as a case study. The results of the predicted values using the recursive and N approaches are presented in Section 3.3, Section 3.4 and Section 3.5, while Section 3.6 is devoted to the discussion.

### 3.1. Daily Numbers of Cases and Deaths

**Figure 3:**
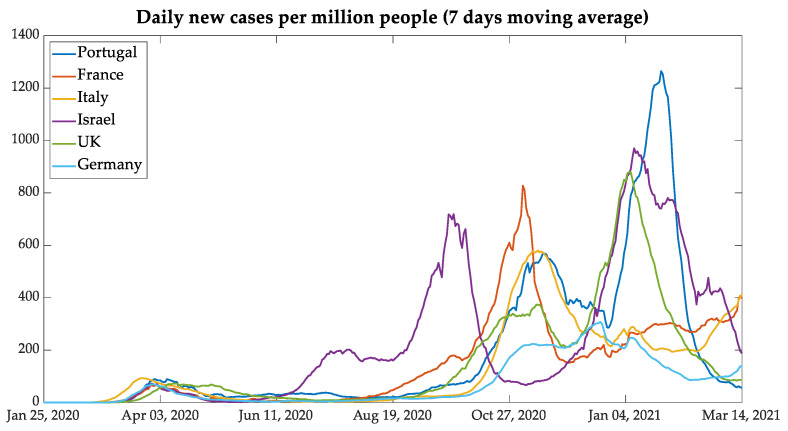
New cases with data normalized per million people.

**Figure 4:**
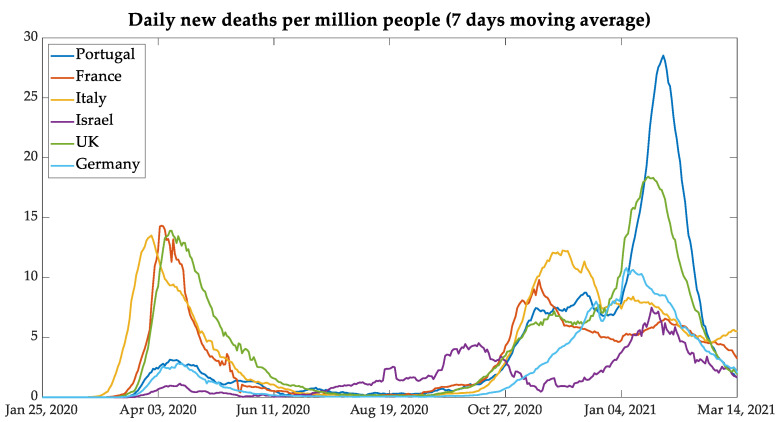
New deaths with data normalized per million people.

After the data were normalized per million people and smoothed with a seven-day moving average, Figure 3 and Figure 4 display the behavior of the contamination and deaths due to COVID-19 as seen in the six countries. It is possible to point out the three waves of daily new infections and deaths of European countries and the four waves in Israel. The waves in European countries are more or less synchronized, but Israel shows a different behavior. The chosen group of countries, composed by the five European countries and Israel, was defined to verify the results of similar mitigation procedures that were adopted by the European Union and the United Kingdom, as well as Israel since this country has a procedure that was more advanced in vaccination.

The similarities among the contagion curves of the European Union and the United Kingdom resulted in mitigation actions taken by mutual agreement, even with the exit of the United Kingdom from European Union in January 2020 (i.e., Brexit).

In a comparative analysis between Portugal and the United Kingdom, it is possible to note similarities in the second and third waves, in which the behavior of daily new cases and deaths was very similar. This fact can be associated with the B.1.1.7 lineage (known as the UK COVID-19 variant, and reclassified as VOC-202012/01Alpha), which showed itself as more contagious than the nonvariant strains, aside from representing 40% of the total Portuguese cases and 60% of the cases in the region of Lisboa e Vale do Tejo until February 2021 [18,19,20]. However, in the next month, the variant reached the mark of 82.9% of Portuguese cases [21]. Another similarity between these countries was the relaxation of contingency rules during the Christmas holidays.

In analyzing the third wave, it can be seen that the countries which adopted lockdown during the months of November and December, such as Germany and France, had less pronounced curves of new daily cases and deaths per million people. One exception is Italy: notwithstanding that it had adopted relaxed measures similar to Portugal and the UK during the holidays, it had no pronounced curves of new cases and deaths.

### 3.2. Israel: A Case Study

The Middle-Eastern country Israel was taken as an important case to study since it has shown itself as very advanced in the vaccination process. From the analysis of data on new cases and deaths presented in Figure 3 and Figure 4, the case of Israel presents a different behavior from that of the European countries also used in the present study. 

**Figure 5:**
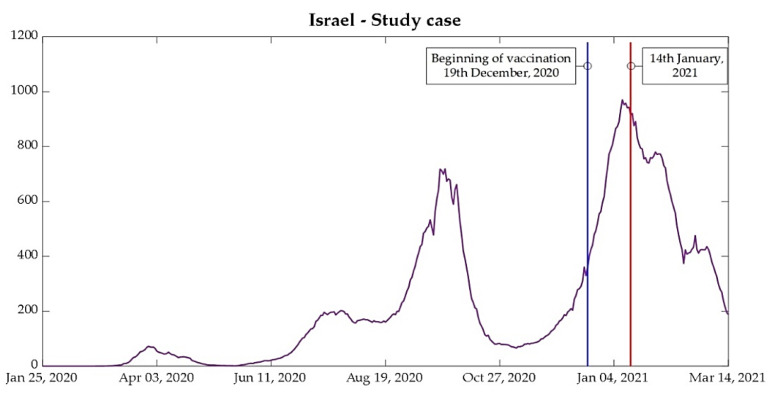
Daily new cases for Israel per million people.

Some points should be noted regarding the behavior of the curves under analysis per million people. Israel had a second wave before the European countries between July and August, and the third wave of contamination occurred between the second week of September and the first week of November, while European countries, except for Germany, had the second wave between the second week of October and the beginning of December. Therefore, Israel was taken out from the joint analysis and was analyzed as an individual case study. The importance of analyzing Israel is due to the highest percentage of people vaccinated in a short period. The start of vaccination in the country began on 19 December 2020, and the country reached 1 million people vaccinated, with the first dose on 1 January 2021 [22,23]. The results of mass immunization can be seen in the curves of new cases, presented in Figure 5, but some considerations are worthwhile.

Vaccination in Israel has been, so far, conducted exclusively through the vaccine produced in a partnership between BioNTech and Pfizer, which proved to be 95% effective in the second and third phase of the clinical study, carried out in terms of randomized, observer-blind, and placebo control [8,22,24,25].

According to the preprint released by Technion (Israel Institute of Technology), the analyses carried out after the start of the vaccination, analyzed between 19 December and 25 February, show a gradual reduction in the rate of infection from 12 days after the application of the first dose and stabilizing on day 35. This resulted in a reduction in the viral load due to vaccines after 12 days, which makes it harder for the virus to infect new individuals [8,25].

Since the virus has an incubation period ranging from 2 to 14 days [9], a drastic reduction in the number of new cases was expected from the second week of January (14 January, labeled in Figure 5), corresponding to 12 days after 1 million people vaccinated, as shown in Figure 5.

Another point is that even after the beginning of the flexibilization of lockdown in the country, adopted on 21 February after 45% of the population vaccinated with the first dose of the vaccine, the curve of new cases continued to reduce, alongside new deaths and ICU patients [23,26].

The advanced vaccination in Israel, conducted exclusively with the BioNTech–Pfizer vaccine, is directly associated with the deal between the government and the American company. According to The Times of Israel, the government paid 43% more than the United States and the European Union for the vaccine. One other relevant point is that Israel agreed to provide data results of mass vaccination, both quantitative and qualitative, according to geographic regions, age, and sex. Furthermore, Israel accepted the responsibility for the vaccine production—the opposite direction taken by European Union—and this demanded the company to be responsible for the vaccine [22].

Since the first week of April 2021, Pfizer company and the Israeli government have been working on a new vaccine supply to continue selling doses for the country, and since the deal agreement, in November 2020, over 8 million doses were already produced [27,28,29,30]. Additionally, even though the vaccination campaign was made exclusively in the country with the BioNTech–Pfizer vaccine, the government still has a deal with Moderna company, whose vaccine was approved to be used since January 2021 [27,29].

### 3.3. Recursive Approach Results

As explained in Section 2.3, the recursive approach makes predictions one day ahead using data of new cases, deaths, and daily ICU patients from twenty-one days earlier, and also the data on vaccination and the wearing of masks one day earlier. This methodology updates the input data in each iteration with predicted values of the previous iteration, creating a vector of twenty-eight days forecasted. Consequently, this approach accumulates errors because it uses predicted values in the ANN input since the second iteration.

The forecasting of new cases, deaths, and daily ICU patients is made in a separate ANNs. Each ANN performs forecasting for the five European countries. Only the predictions for the 28 days of the test set are considered in further analysis. All the presented data are normalized per million people.

The results of Portugal and the UK were analyzed together, considering that the B 1.1.7 COVID-19 variant already represents 82.9% of the Portuguese nationwide cases [21]. In spite of this, the mass vaccination campaign in the UK is showing itself to be faster than the Portuguese campaign, resulting in a divergence of real and forecasted values [31].

**Figure 6:**
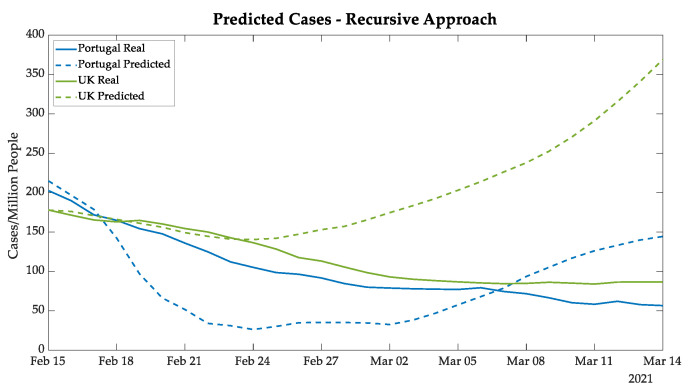
New cases predicted for Portugal and the UK using the recursive approach.

Figure 6 presents Portuguese new cases in blue, and it is possible to note that the ANN predicted an abrupt decrease for Portugal, followed by an increase in new daily cases, which can be explained by the training data. The model predicted a new wave similar to the new wave after the second wave. It is possible that this new wave was avoided by the strong lockdown after 22 January. For the world data, this new wave started in mid-February [9].

On the other hand, for the UK cases (shown by green curves in Figure 6, the gap between the real and predicted cases for the UK started after 24 February. The divergence presented in the plot is justified by the quick vaccination in the UK, which reached more than 30% of the population vaccinated with the first dose of the vaccine on 1 March. It is important to highlight that so far, the UK has been using the BioNTech–Pfizer and AstraZeneca vaccines with efficiencies of 95% and 76%, respectively [8,32,33,34,35].

**Figure 7:**
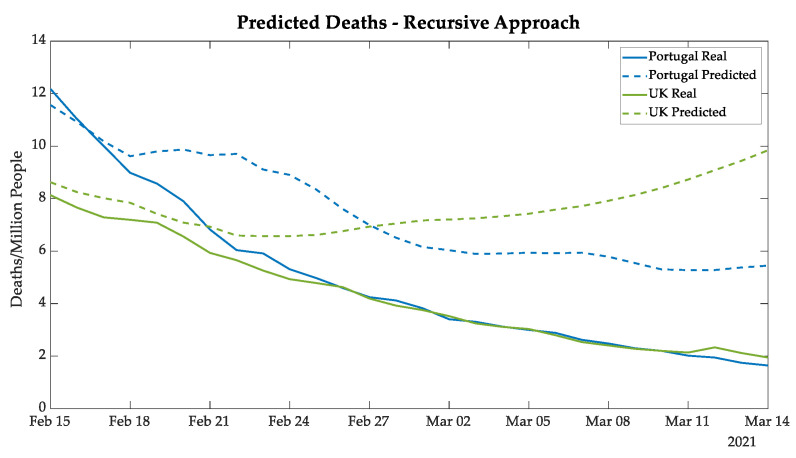
New deaths predicted for Portugal and the UK using the recursive approach.

**Figure 8:**
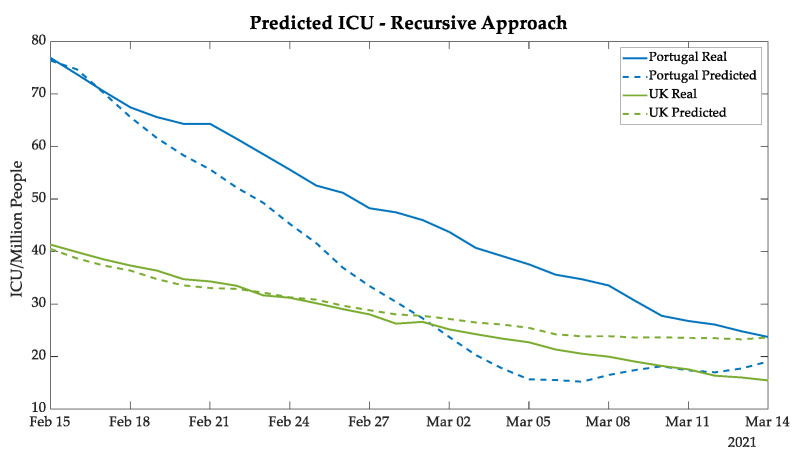
Daily ICU patients predicted for Portugal and the UK using the recursive approach.

The deaths and ICU patients’ forecasting results of Portugal followed the same tendency as the real data, as shown in Figure 7 and Figure 8, demonstrating a good prediction until 18 February and then satisfactory results for the next 24 days.

The gap tendency—as verified in the UK new cases curves—between real and predicted values was also verified in the curves for deaths and ICU patients, as also demonstrated in Figure 7 and Figure 8.

**Figure A1:**
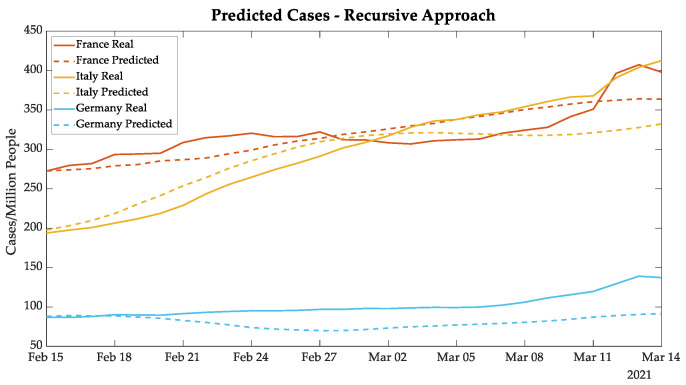
France, Italy, and Germany daily new cases using the recursive approach.

**Figure A2:**
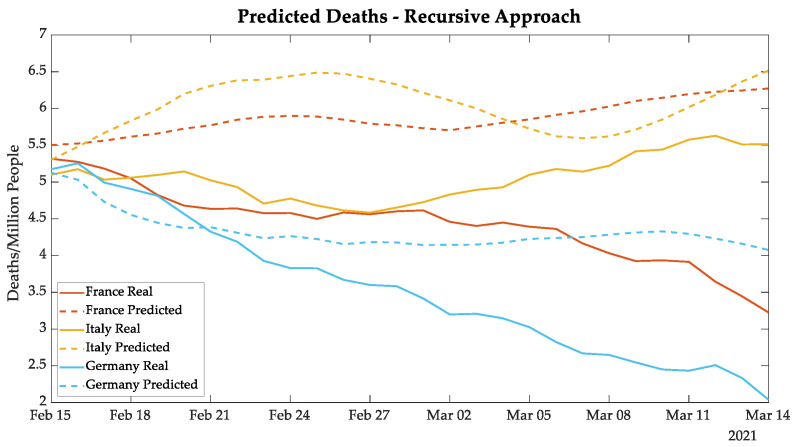
France, Italy, and Germany daily deaths using the recursive approach.

**Figure A3:**
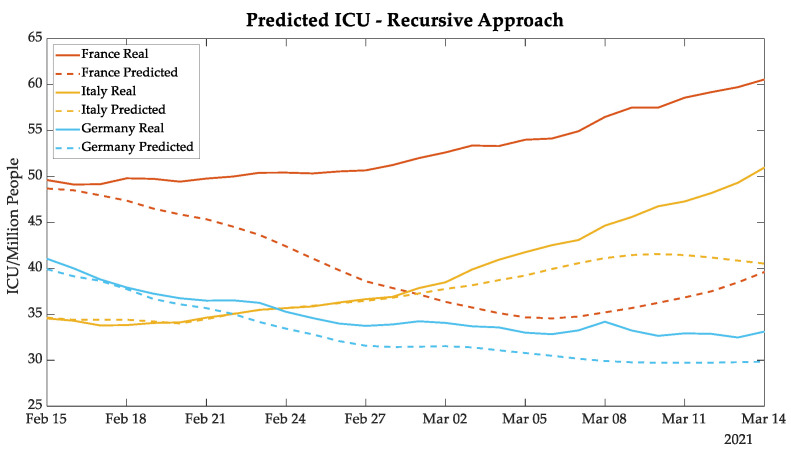
France, Italy, and Germany daily ICU patients using the recursive approach.

Figure A1, Figure A2 and Figure A3 in Appendix A show the other countries’ prediction of new cases, deaths, and ICU patients, by using the recursive approach. The best results were defined by the analyses of the MAE in order to verify the best ANN topology.

### 3.4. N Approach Results

The second approach to predict new cases, deaths, and daily ICU patients had a methodology that used the same input data as the previous one, but the forecasting values were defined directly by the predictions of n, n + 7, n + 14, and n + 21 days, which were used to create a vector of seven days ahead each.

**Figure 9:**
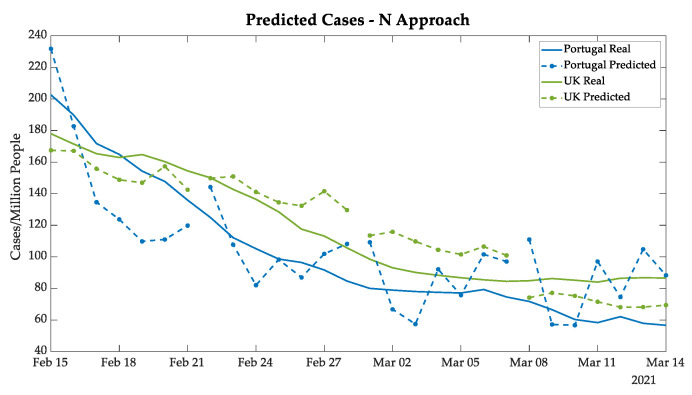
New cases predicted in Portugal and the UK using the N approach.

The greatest advantage of the N approach is that it does not accumulate errors by iteration, since the prediction is directly obtained from real data. However, since it used a limited database, the main error source came from the values of the daily variation, even with the moving averages. The tendency of new cases in Portugal and in the UK, shown in Figure 9, had a significant variation among days, as expected due to the variation in the training data. Nevertheless, the UK prediction behaves quite well.

**Figure 10:**
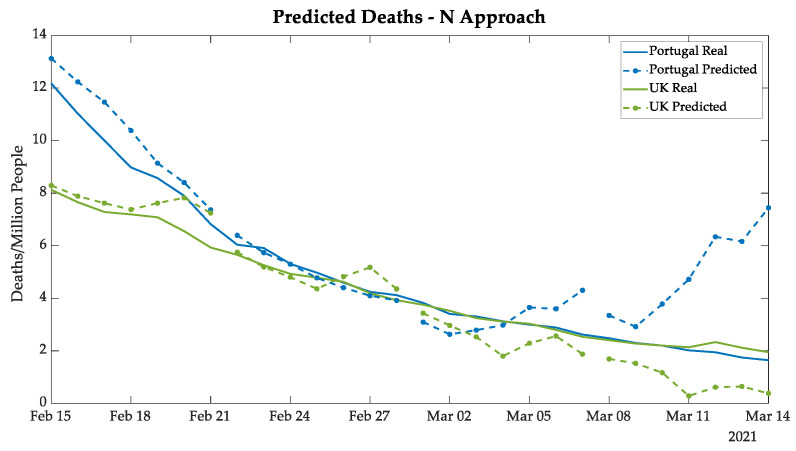
New deaths predicted in Portugal and the UK using the N approach.

**Figure 11:**
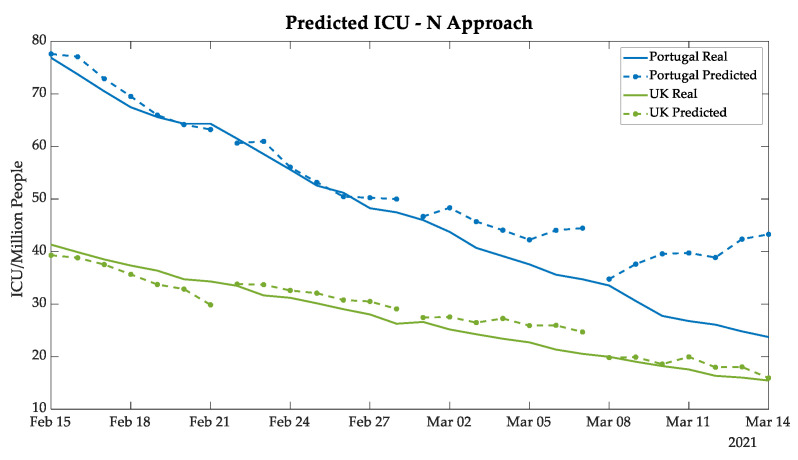
Daily ICU patients predicted for Portugal and the UK using the N approach.

In Figure 10 and Figure 11, the plots demonstrate highly satisfactory results in the deaths and the ICU patients forecasting, respectively, for both Portugal and the UK. The absolute errors increase substantially only in n + 21 analysis, which can be explained by the distance between the predicted values and the real data known by the ANN. An additional point is that the tendency of deaths followed the ICU patients, which is expected since the ICUs represent the serious cases of COVID-19, and unfortunately, at times those cases resulted in death.

Analyzing the UK prediction, in Figure 9, Figure 10 and Figure 11 the results of the UK demonstrate that the N approach was better in forecasting than the recursive approach, considering that it followed the tendency of new cases, deaths, and daily ICU patients, without a substantial gap as demonstrated in the previous approach. The comparative analyses of the two approaches are discussed in Section 3.5.

**Figure A4:**
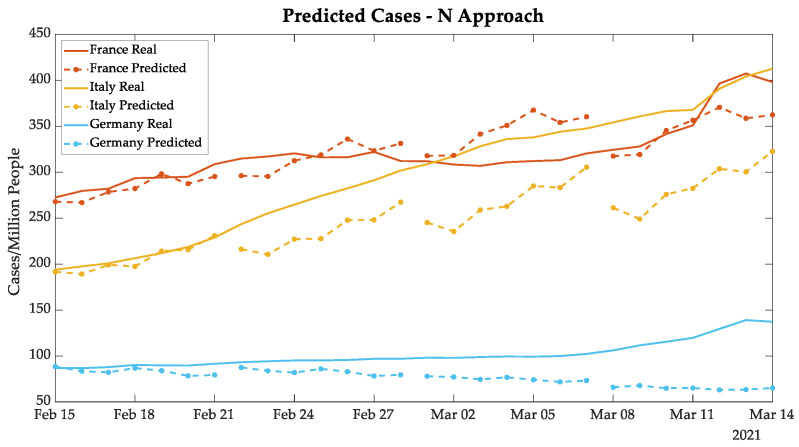
France, Italy, and Germany daily new cases using the N approach.

**Figure A5:**
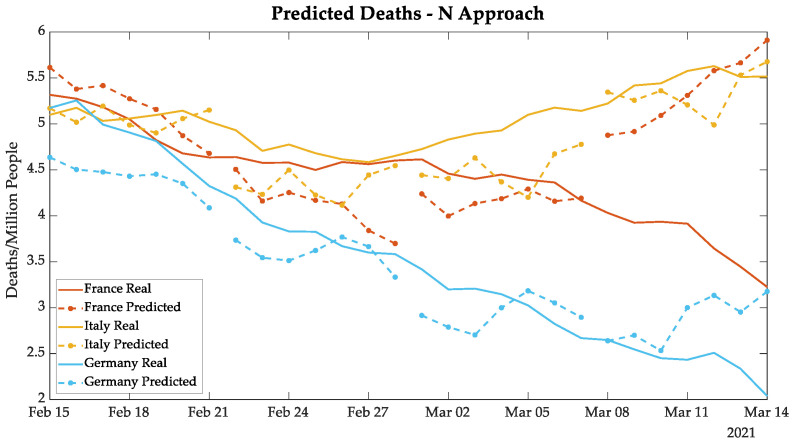
France, Italy, and Germany daily deaths using the N approach.

**Figure A6:**
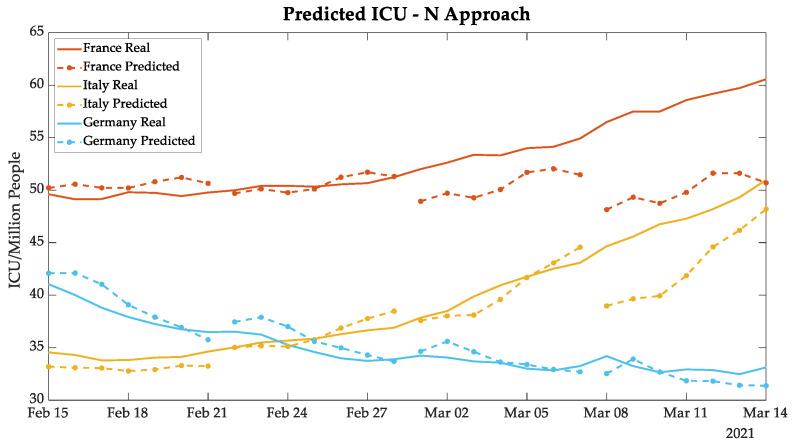
France, Italy, and Germany daily ICU patients using the N approach.

The plots that show the predicted values of France, Italy, and Germany are presented in Appendix B, Figure A4, Figure A5 and Figure A6.

### 3.5. Comparison of Forecasting Approaches

The two proposed forecasting methodologies were analyzed by the MAE over the test set (1 to 28 days ahead) for the 5 European countries, for the 3 parameters (cases, deaths, and ICU patients), as presented by Table 3. The MAE is presented for each week ahead and for the whole 4 weeks ahead, and also as an absolute measure of the mean error per million people. Results are presented by country and for all five countries (i.e., “Total” in Table 3).

For the number of cases, the predictions made by each approach are analyzed along with the five countries. Generally, it can be observed that the N approach made better forecasting than the recursive approach for Portugal, France, and the UK, in spite of behaving worse predictions for Italy and Germany, considering the four weeks together (n: n + 27) and also for the long term prediction (fourth weeks ahead, n + 21: n + 27). Multiplying the predicted number by the population of the country, the long-term prediction (4 weeks ahead) is about 930 daily cases for the UK (lower MAE), 5670 for Italy (higher MAE), and 260 for Portugal. Regarding the MAE in all five countries, the N approach clearly made a better prediction, with an MAE lower than 30 cases per million people.

Concerning numbers of deaths, the N approach made better forecasting than the recursive approach for the five countries. The MAE varied between 0.3 to 1.2 daily deaths per million people for the whole 4 weeks ahead. In the forecasting for only the long term (i.e., 4 weeks ahead), the MAE varied between 0.22 for Italy and 2.91 for Portugal, considering that the whole population of the country is a daily MAE variation between 13 deaths in Italy and 29 in Portugal for the long-term forecasting. Regarding the MAE in all five countries, the N approach had a more satisfactory prediction, with an MAE lower than 0.6 deaths per million people.

For the ICU patients prediction, the N approach behaved better than the recursive approach for the five countries. The MAE varied between 0.94 for Germany and 5.03 for Portugal per million people for the whole 4 weeks ahead. The long-term prediction presented an MAE between 1.04 for Germany and 11.84 for Portugal. For the whole population of the country, this represents an MAE between the total number of 84 ICU patients in Germany and 118 ICU patients in Portugal. With respect to the MAE in the five countries, the N approach also received a better prediction, with an MAE lower than 2.5 ICU patients per million people.

After verifying that the N approach shows itself as a better methodology in absolute numbers, Table 4 presents the relative number of errors, calculated based on forecasted and real values. The MAPE indicates the increase of the relative error proportionally to the space between the real data, acknowledged by the ANN, and the forecasted values.

It is important to highlight that a high percentage error can also be associated with the scale under evaluation. The biggest percentage errors presented in Table 4 is related to deaths, which represent the smallest predicted values. The relative measure of MAPE needs to be read carefully, considering that for low real values, a small absolute error may induce a very high value of MAPE, as it is the case for the number of deaths in the fourth week ahead.

**Figure 12:**
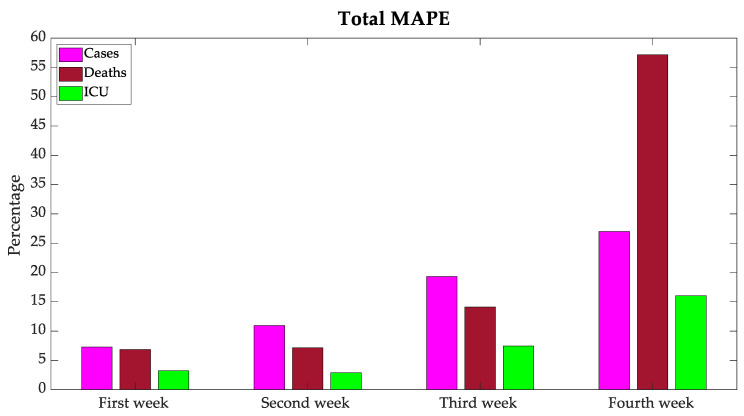
Total mean absolute percentage error.

The graphic representation of total MAPE for all the countries under study is shown in Figure 12. As it was expected, the further away the forecast, the greater the MAPE.

### 3.6. Discussion

Based on the analysis of the prediction made by the two approaches, an improved prediction made by the N approach for the prediction of the deaths and ICU patients is clear. For the number of cases, the N approach also behaved better for 3 of the 5 countries. Therefore, this architecture of prediction demonstrated an advantage over the recursive approach. This may be due to the error accumulation from day 2 onward in the recursive approach.

The use of ANN to predict the number of cases and deaths for the next 6 [4], 7 [5], 10 [6], and 40 [7] days was already experimented but for accumulative values, with promising results using a recursive approach and a variety of countries. The results of the present work cannot be compared to results of previous similar works because the relative error of the accumulative cases cannot be directly compared with the absolute error of daily predictions.

Concerning the MAE of the forecasting for the five European countries, Portugal presents the higher error in the predicted number of deaths and ICU patients, possibly because of the strong decay caused by the strong lockdown just before the term that was used for the test set. The values of cases deaths and ICU patients for this country suffered an abrupt increase caused by relaxed rules during the Christmas holidays. These similar tendencies were observed in large Eurasian nations such as India and Russia with a rather massive pool of vaccinated citizens during the same term [36,37,38].

It was expected that the virus propagation followed the same lead for all the countries under study; however, due to the variants and different severity of contingency rules, the plots present a different perspective. As shown in previous sections, the behavior of new cases and deaths in Portugal and the UK are similar, as explained by the B 1.1.7 variant being more infectious [39].

Another association possible to be made is between France and Germany since both countries adopted a restricted lockdown during November and December 2020 [40], which resulted in an attenuated third wave. Even in small island states such as Malta, comparable challenges were observed [41].

## 4. Conclusions

The data analysis presented in this paper proposed a review of the three waves of COVID-19 in European countries in association with mitigation procedures. The main goal was to compare the vaccination campaigns and the results according to new infections, deaths, and ICU patients. In the first tests, Israel was considered as part of the study group compound, but because the infections in the country presented a different behavior compared to the other countries under analyses, it was eventually taken out and became a separate case study.

From this work, it is possible to analyze coronavirus propagation waves, considering the mitigation procedures and vaccination campaigns. An additional goal of this study was to investigate the data normalized per million people in the country, independent of the size of the country. The results show the efficiency of a restricted lockdown in decreasing the virus propagation and most of all, the efficiency of a massive vaccination campaign, following the WHO’s recommendation.

The experimented models used to predict the number of new daily cases of infection, deaths, and ICU patients were based on two approaches. The recursive approach made the prediction for the next day in a recursive way until 28 days ahead. The N approach made the prediction for one day of the next four weeks and recursively determined the other 6 days of each week. One ANN was used for each parameter—i.e., cases, deaths, and ICU patients—due to the delay of these time series, succeeding the sequence of cases followed by ICU patients and later deaths. The ANN has the previous 21 day’s numbers of cases, deaths, and ICU, plus the wearing of masks and the percentage of population with one dose of a vaccine at least, and the country identification, in its input. The ANN models made predictions for the next day until 28 days ahead.

The N approach presented improved forecasting performance for the 3 variables and was selected as the best approach model. The MAE for all the terms of the next 28 days for the five countries (Portugal, France, Italy, the UK, and Germany) was lower than 30 cases, 0.6 deaths, and 2.5 ICU patients per million people, and the MAPE was lower than 16.14% of cases, 21.32% of deaths, and 7.40% of ICU patients. The hardest forecasting for the long-term analysis of 4 weeks ahead only behaved with an MAE below 51 cases, 1.2 deaths, and 5.4 ICU per million people, and an MAPE lower than 26.99% of cases, 57.16% of deaths, and 16.04% of ICU patients. These final results can be considered at the level of a reasonable forecasting to be considered by decision makers about eventual future restrictions.

## Figures and Tables

**Table 1:** Best ANN topology using recursive approach.

Predicted Data	HL No. of Neurons	HL Function	OL Function
Cases	4	Elliot	Linear
Deaths	13	Elliot	Linear
ICU	13	Elliot	Linear

HL: hidden layer, OL: output layer.

**Table 2:** Best ANN topology using the N approach.

Predicted Data	HL No. of Neurons	HL Function	OL Function
Cases	4	Elliot	Linear
Deaths	18	Elliot	Linear
ICU	15	Elliot	Linear

HL: hidden layer, OL: output layer.

**Table 3:** Mean absolute error comparative analyses.

	Recursive Approach			N Approach		
	Cases	Deaths	ICU	Cases	Deaths	ICU
Portugal n: n + 6	38.83	1.08	3.18	30.29	0.95	1.45
Portugal n + 7: n + 13	69.50	3.14	12.29	12.92	0.18	1.37
Portugal n + 14: n + 20	28.16	2.80	20.29	17.52	0.75	5.44
Portugal n + 21: n + 27	60.87	3.38	10.02	25.98	2.91	11.84
Portugal n: n + 27	49.34	2.60	11.44	21.68	1.20	5.03
France n: n + 6	10.23	0.63	2.35	8.18	0.20	1.03
France n + 7: n + 13	14.44	1.27	9.39	13.03	0.47	0.46
France n + 14: n + 20	21.86	1.41	18.00	32.41	0.24	3.03
France n + 21: n + 27	27.02	2.44	21.42	19.34	1.60	8.51
France n: n + 27	18.39	1.44	12.79	18.24	0.63	3.26
Italy n: n + 6	13.79	0.74	0.26	4.07	0.12	1.10
Italy n + 7: n + 13	18.99	1.71	0.07	38.29	0.37	0.61
Italy n + 14: n + 20	14.88	0.90	1.84	63.37	0.47	0.85
Italy n + 21: n + 27	56.70	0.57	6.38	94.47	0.22	4.77
Italy n: n + 27	26.09	0.98	2.14	50.05	0.30	1.83
UK n: n + 6	3.76	0.62	1.17	10.18	0.58	2.10
UK n + 7: n + 13	20.84	1.96	0.72	12.32	0.60	1.82
UK n + 14: n + 20	104.65	4.24	2.43	17.97	0.65	3.04
UK n + 21: n + 27	211.18	6.59	6.08	13.75	1.30	1.13
UK n: n + 27	85.11	3.35	2.60	13.56	0.71	2.02
Germany n: n + 6	2.99	0.22	0.63	6.12	0.44	1.16
Germany n + 7: n + 13	21.82	0.42	1.96	12.47	0.25	1.01
Germany n + 14: n + 20	23.72	1.12	2.53	24.30	0.31	0.56
Germany n + 21: n + 27	36.21	1.82	3.28	57.60	0.46	1.04
Germany n: n + 27	21.19	0.89	2.10	25.12	0.37	0.94
Total n: n + 6	13.90	0.67	1.31	10.49	0.40	1.32
Total n + 7: n + 13	27.43	1.70	4.08	21.22	0.37	0.98
Total n + 14: n + 20	34.69	1.90	7.82	36.49	0.48	2.30
Total n + 21: n + 27	74.78	2.56	8.93	50.94	1.12	5.34
Total n: n + 27	37.70	1.71	5.54	29.78	0.59	2.49

**Table 4:** Mean absolute percentage error for the N approach.

	MAPE for N Approach		
	Cases	Deaths	ICU
Portugal n: n + 6	18.62	10.01	2.06
Portugal n + 7: n + 13	12.93	3.66	2.63
Portugal n + 14: n + 20	22.44	24.70	14.46
Portugal n + 21: n + 27	42.58	157.18	45.68
Portugal n: n + 27	24.14	48.89	16.21
France n: n + 6	2.81	4.08	2.09
France n + 7: n + 13	4.13	10.26	0.92
France n + 14: n + 20	10.37	5.45	5.66
France n + 21: n + 27	4.99	44.59	14.54
France n: n + 27	5.57	16.09	5.80
Italy n: n + 6	1.98	2.44	3.21
Italy n + 7: n + 13	14.04	7.76	1.67
Italy n + 14: n + 20	19.25	9.42	2.07
Italy n + 21: n + 27	24.98	4.01	10.15
Italy n: n + 27	15.06	5.91	4.27
UK n: n + 6	6.19	8.75	5.75
UK n + 7: n + 13	10.62	7.60	6.27
UK n + 14: n + 20	20.12	21.08	13.45
UK n + 21: n + 27	16.02	60.00	6.69
UK n: n + 27	13.24	24.36	8.04
Germany n: n + 6	6.83	8.92	2.98
Germany n + 7: n + 13	13.04	6.53	2.87
Germany n + 14: n + 20	24.43	9.96	1.67
Germany n + 21: n + 27	46.40	20.03	3.14
Germany n: n + 27	22.67	11.36	2.67
Total n: n + 6	7.28	6.84	3.22
Total n + 7: n + 13	10.95	7.16	2.87
Total n + 14: n + 20	19.32	14.12	7.46
Total n + 21: n + 27	26.99	57.16	16.04
Total n: n + 27	16.14	21.32	7.40

## Data Availability

The data about the time series of the number of cases, deaths, and ICU patients, used in the research, were extracted from https://www.worldometers.info/coronavirus/?utm_campaign=homeAdUOA?Si (accessed on 16 March 2021); the data about vaccination were extracted from https://ourworldindata.org/covid-vaccinations (accessed on 16 March 2021); the wearing of masks data were extracted from: https://masks4all.co/pt/what-countries-require-masks-in-public/ (accessed on 16 March 2021).

## Appendix A

Results from the recursive approach in predicting daily new cases, deaths, and ICU patients to France, Italy, and Germany.

## Appendix B

Results from the N approach in predicting daily new cases, deaths, and ICU patients for France, Italy, and Germany.
